# A novel sideways fall simulator to study hip fractures ex vivo

**DOI:** 10.1371/journal.pone.0201096

**Published:** 2018-07-24

**Authors:** Ingmar Fleps, Muriel Vuille, Angela Melnyk, Stephen J. Ferguson, Pierre Guy, Benedikt Helgason, Peter A. Cripton

**Affiliations:** 1 Institute for Biomechanics, ETH Zürich, Zürich, Switzerland; 2 Orthopaedics and Injury Biomechanics Group, Department of Mechanical Engineering and Orthopaedics and School of Biomedical Engineering, University of British Columbia, Vancouver, Canada; 3 Division of Orthopaedic Trauma, Department of Orthopaedics, University of British Columbia, Vancouver, Canada; 4 School of Science and Engineering, Reykjavik University, Reykjavik, Iceland; Universidad de Zaragoza, SPAIN

## Abstract

Falls to the side are the leading cause of hip fractures in the elderly. The load that a person experiences during a fall cannot be measured with volunteers for ethical reasons. To evaluate injurious loads, while considering relevant energy input and body posture for a sideways fall, a subject-specific cadaveric impact experiment was developed. Full cadaveric femur-pelvis constructs (N = 2) were embedded in surrogate soft tissue material and attached to metallic surrogate lower limbs. The specimens were then subjected to an inverted pendulum motion, simulating a fall to the side with an impact to the greater trochanter. The load at the ground and the deformation of the pelvis were evaluated using a 6-axis force transducer and two high-speed cameras. Post-test, a trauma surgeon (PG) evaluated specimen injuries. Peak ground contact forces were 7132 N and 5641 N for the fractured and non-fractured specimen, respectively. We observed a cervical fracture of the femur in one specimen and no injuries in a second specimen, showing that the developed protocol can be used to differentiate between specimens at high and low fracture risk.

## Introduction

Among fragility fractures, hip fracture is the most devastating type. [1, 2] They are associated with a high risk of morbidity [3, 4] and mortality, [5, 6] and an immense financial burden on the health care system. [7] State of the art screening for hip fracture risk is based on areal bone mineral density (aBMD). However, more than 50% of hip fractures occur in people that are not identified by this metric. [8–10] An alternative metric for femur is quantitative computed tomography (CT) based finite element analysis (FEA). [11–13] Although this metric performs better than aBMD against cadaveric femoral strength measures, in cohort studies, FEA-based femoral strength prediction demonstrated only marginal improvement over aBMD-based risk assessment, [14–23] suggesting that both metrics may not account for all relevant biomechanical factors. Another important aspect affecting the risk of hip fracture is the load experienced by the femur due to a fall. [24–27] The soft tissues padding the greater trochanter were reported to attenuate the force transferred through the femur by 71 N per millimetre of soft tissue thickness. [28] Moreover, the load transferred through the femur was reported to be about 20% lower than the load measured at the impact surface. [29] Previous cadaveric studies focused on the femur [12, 30–32] or placed the femur-pelvis construct into boundary conditions that simulated car crash scenarios. [33–35] Fall simulators that modelled the pelvic compliance [32, 36] used springs with characteristics based on the average compliance of the whole pelvic region at low force levels, thus neglecting subject specific variability in pelvic compliance and non-linearity of the pelvic response. State of the art models to predict the impact load resulting from a fall used single degree of freedom (sDOF) spring-mass-dashpot models. [26, 37] These models were informed by studies with human volunteers falling onto soft surfaces, [38, 39] or from low height. [24, 27] Further input was provided based on multi-body dynamics models. [26, 37] The sDOF models were optimized to predict the peak load of the validation sets. The data sets from volunteers were small and experiments were limited to non-injurious loads for ethical reasons. These models were then extrapolated to more severe loads, neglecting the non-linearity of the compliance of pelvic region. [24, 40] Furthermore, these sDOF models did not represent the force-time response of the impacts accurately. A combination of the sDOF models and dual energy x-ray absorption (DEXA) based FEA to predict femoral strength have been used to assess fracture risk. [41] These studies showed that incorporating the prediction of impact loads can affect the fracture risk prediction.

The goal of this study was to develop a dynamic inertia-driven sideways fall protocol that allows for testing of full cadaveric femur-pelvis constructs under realistic boundary conditions. Further, we aimed to impact specimens in an alignment representative of a sideways fall with the goal to create femoral fractures and not pelvic fractures. Furthermore, we suspected that using realistic kinetic energy and mass moments of inertia would result in clinically relevant fracture and non-fracture outcomes. Finally, we postulated that the influence of pelvic compliance on force-time response of the system up to failure can be effectively evaluated in an impactor experimental model of this type.

## Materials and methods

### Specimen preparation

The study was approved by the University of British Columbia Clinical Research Ethics Board (Study ID H06-70337). Specimen preparation and experiments were conducted at the UBC Centre for Hip Health and Mobility and the International Collaboration On Repair Discoveries (ICORD) centre. Two fresh frozen cadaveric specimens (1 male, 1 female) were obtained from a tissue bank (ScienceCare, Phoenix, AZ, USA). Consent was given either written or electronically by the donor or next of kin. The donors had no history of skeletal fractures of the femurs or pelvis. Skin, fat, muscle, and intestines were removed during dissection, leaving only bone, cartilage, and ligaments. Both femurs were cut 175 mm below an estimated greater trochanter impact point. The distal ends of the femurs were plugged to contain the marrow. The L5 vertebra and all tissues superior to it were removed, while keeping the sacroiliac ligaments intact from the base of sacrum downward. Specimens were kept hydrated by spraying them with saline solution or wrapping them in saline-soaked rags. Specimens were stored in a freezer at -20C when not being prepared.

**Fig 1:**
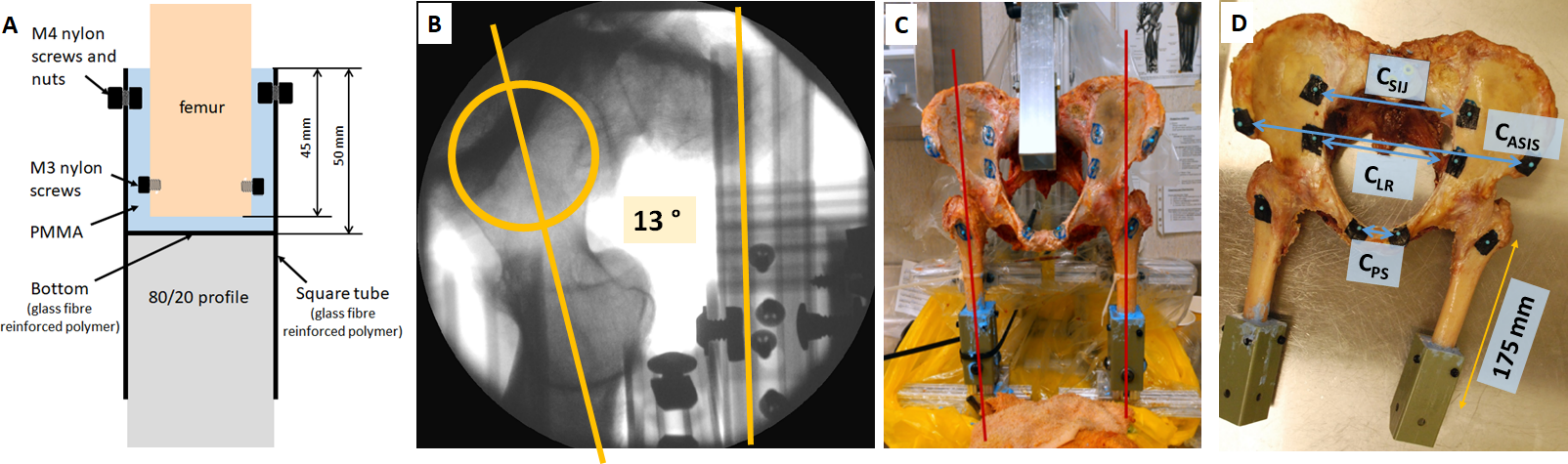
Specimen alignment. (A) Schematic of the femoral potting method using a square tube, PMMA and polymer screws, (B) C-arm image taken at a 20° angle to an axial view to align the femur to 13° anteversion. The estimated femoral neck axis and reference profile for the simulated condyle tangent are shown in orange. (C) Dissected specimen in custom alignment rig with distal femurs being set in tubes with PMMA. The red lines schematically show the laser used for alignment. (D) Specimen with femoral fixations (green tubes) and sacral fixation holes. The distance used to cut the femurs is shown in orange. The deflection between markers that was evaluated during the impact are drawn in blue. *d_PS_* was used to monitor the deflection across the pubic symphysis; *d_PB_* was used to monitor the deflection across the pelvis, close to the acetabular cups; *d_SIJ_* was used to monitor the deflection across the sacrum; *d_ASIS_* was used to monitor the deflection between the anterior superior iliac spines as a measure out of plan of the pelvic brim.

Specimen alignment was controlled by fixing both femurs with respect to square tubes using poly methyl-methacrylate (PMMA). A custom alignment rig and laser levels were used to align the femoral shaft parallel to the long axis of the tubes in the coronal and sagittal plane. A 13° anteversion angle was chosen for both femurs. [42] This angle was set based on x-ray images (Fig 1). The femoral neck alignment with respect to the square tube was used as a surrogate measure for femoral anteversion. The alignment in the setup was carefully documented prior to each drop using a digitizing probe (Optotrak Certus, Northern Digital Inc., Waterloo, ON, Canada). The posture was based on experiments with volunteers, [43] multi-body simulations [37], and anatomical literature. [44, 45] The greater trochanter impact point was adjusted to the same X-Y plane as the foot point. Hip flexion was set to be 37.6°. A pelvic tilt of 12° and a pelvic rotation in the coronal plane (X-Y plane) of 15° was adjusted. Detailed information on alignment angles, including schematics and measured alignment is provided in the appendix (section: target alignment). During pre-tests (n = 6), femoral alignment angles were controlled to an accuracy of < 1 degree and pelvic angles to < 2 degrees. Changes in the alignment of the impacted femur and the pelvis over the decent were smaller < 1 degree. No discontinuity in specimen alignment was recorded when the specimen slipped off the guides.

Clinical resolution CT scans (Toshiba Aquilion One, Ōtawara, Tochigi, Japan) (120 kVp, 200 mAs, voxel size: 0.78 mm X 0.78 mm X 0.3 mm, slice thickness 0.5 mm) were acquired for both specimens after mechanical fixations and pelvic fiducial markers were attached. All specimens were scanned with a hydroxyapatite calibration phantom (QC1, Scanco Medical, Brüttisellen, Switzerland) for conversion of grey scale values to bone density. 3D geometries of markers, bones, and tubes were segmented from CT images using open source software (MITK-GEM, https://simtk.org/projects/mitk-gem). [46] A relationship between pelvic landmarks and marker positions was established and used to estimate the pelvic alignment prior to the experiment. A commercial tool (QCT Pro, Mindways Software, Inc., Austin, Texas) was used to calculate a CT based aBMD.

### Soft tissue surrogate

**Fig 2:**
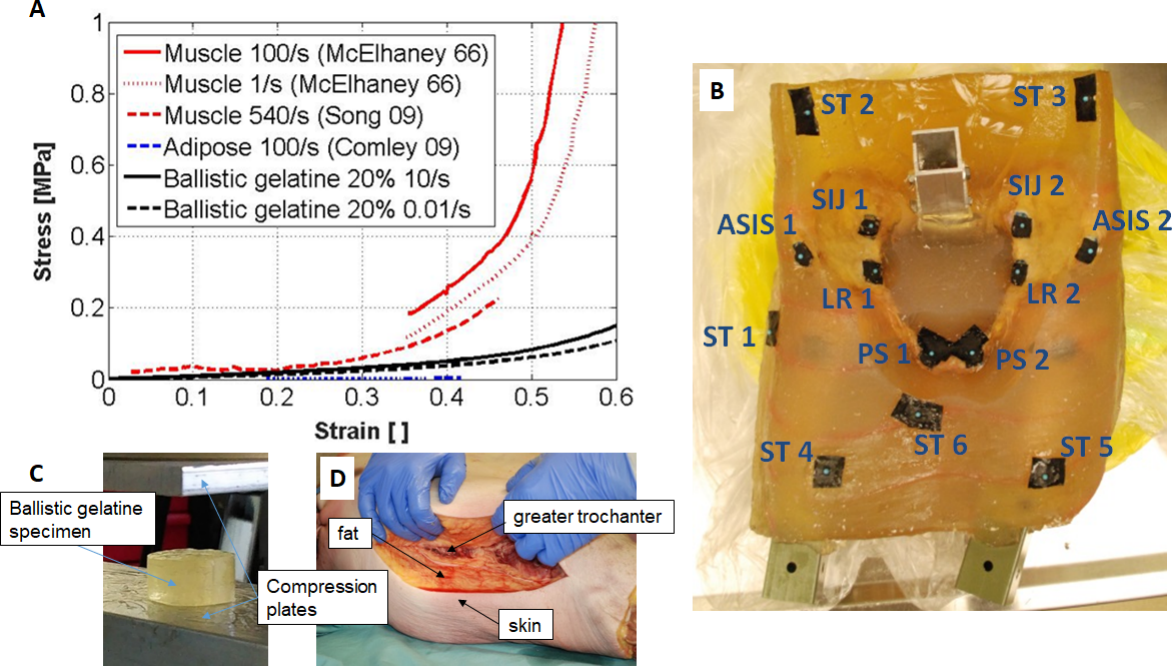
Soft tissue surrogate. (A) Compressive properties of the soft tissue surrogate (20 wt-% ballistic gelatine at room temperature) compared to fat [49] and muscle [47, 48]. (B) Anterior view of embedded specimen (H1391) with fiducial markers for motion tracking. (C) Setup for ballistic gel compression testing and (D) specimen with incision at the greater trochanter, showing the tissue composition over the greater trochanter.

Ballistic gel (Custom collagen®, Addison, IL, USA) with a gelatine content of 20 wt-% was chosen to represent muscle, fat, and other soft tissues in the pelvic region. The mechanical properties of the gel at room temperature were characterized in compression to verify that the response was within the range of reported values for muscle [47, 48] and fat [49] (Fig 2). Cylindrical ballistic gelatine specimen (D = 20 mm, h = 10 mm) were tested at a strain rate of 0.01/s and 10/s. The top and bottom surfaces were lubricated (KY Jelly, Durex, UK) before testing to minimize friction at the compression plates. Five samples were tested up to 80% strain for each strain rate on a mechanical testing device (Dynamight, Instron, Norwood, MA, USA). The mechanical properties of the gel were found to be in between the compressive properties of adipose and muscle tissue. Moulds for generating subject-specific soft tissue geometries were manufactured from polystyrene. Subject-specific surface shapes of the pelvic region were derived from a body shape database (SizeUSA, [TC]^2^ Labs, Apex, NY, USA), based on skeletal geometry, donor height, and body mass index (BMI). Different relationships between BMI and soft tissue thickness over the greater trochanter were used for males and females to calculate target values. [50] A detailed description of the moulding protocol is reported in the appendix (section: soft tissue surrogate).

### Fall simulator

**Fig 3:**
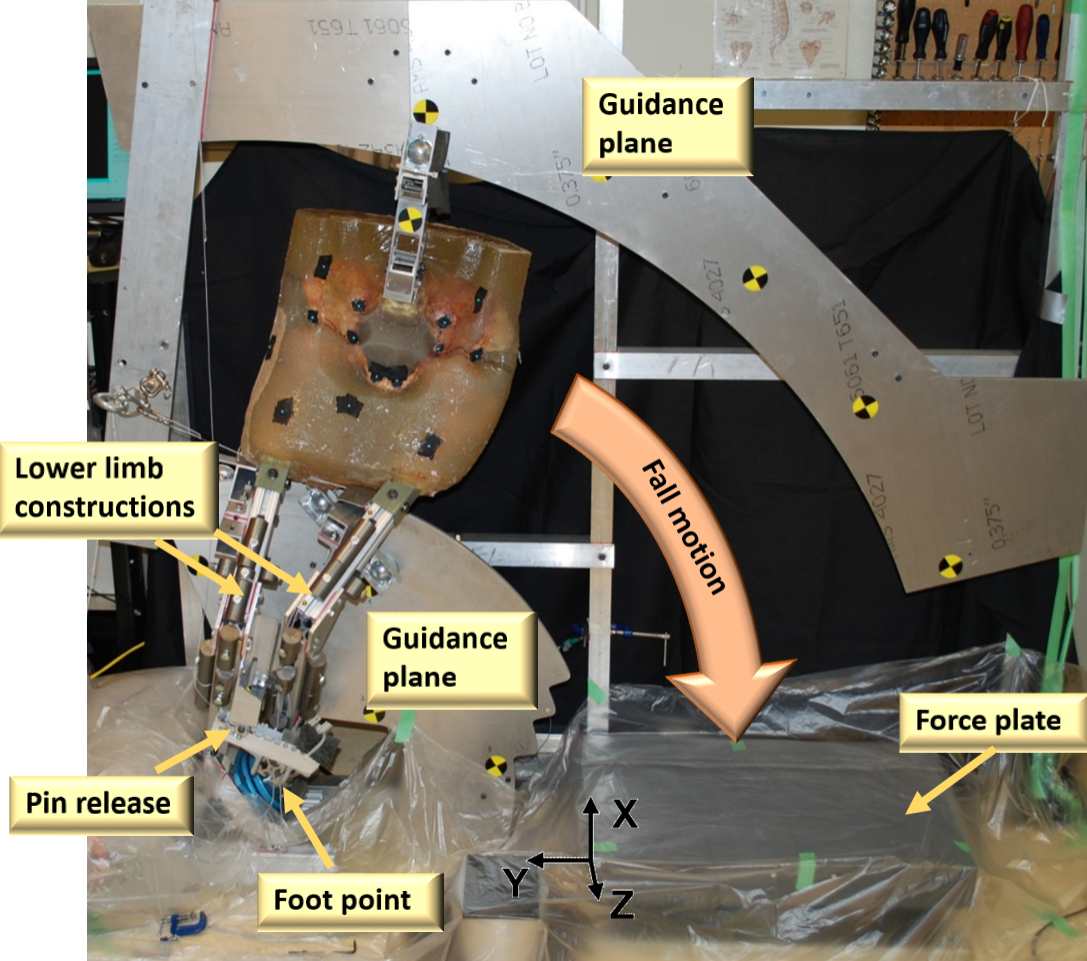
Pendulum setup. Two planes were used to guide rollers attached to the lower limbs and pelvis. These guides constrained the specimen during the *fall phase* to a single degree of freedom (rotation at the foot point using a ball and socket joint and guidance planes). Cut guidance planes allowed for a free impact of the specimens, which slipped off the guidance planes right before impact and were only constrained by the ball and socket joint at the foot point. A pin-release constrained the motion of the contralateral leg during the *fall phase* but released it before impact. Rigid steel masses were added to the lower limb constructs to achieve target limb masses [45]. The knee impact point served to catch the knee at force plate level. Knee impact occurred well after greater trochanter impact.

The fall simulation protocol comprised a two-stage experiment. Stage 1 is the *fall phase* and is defined by a gravity-driven inverted pendulum-style fall with only one rotational degree of freedom about an axis through the foot point. Stage 2 is the *impact phase* and is defined by controlled initial conditions, such as velocity and alignment, and an unconstrained impact. Constraints on the lower limbs and pelvis were released at the end of the fall phase prior to impact. Only the ball and socket joint at the foot point of the impacted leg remained translationally fixed to the ground during the impact phase. The reference coordinate system was based on the force transducer and located at the left frontal corner of the force plate (Fig 3).

A target impact energy based on an impact velocity of 3 m/s [38, 43] and an effective mass (mass contributing to the impact) of 38% of body mass [24, 37, 40] was chosen for this protocol. The resulting kinetic energy translated to an equivalent pendulum inertia and impact velocity (Appendix: Energy calculation). The pendulum height was set independently of subject height to allow for repeatable impact velocities across specimens. A greater trochanter height of 0.760 m in a flexed leg position was chosen for the apparatus, which corresponded to a virtual body height of 1.99 m. The lower limbs were represented by a combination of aluminium profiles and steel masses (Appendix: Lower limb construction). A ball and socket joint was used to fix the rotational point of the pendulum. The masses of all body segments were specimen-specific (Appendix: Mass adjustment). [45] Human cadaveric pelvic-femur constructs were embedded into surrogate soft tissue material (Table 1). The upper body, superior to the navel, was not modelled.

**Table 1:** Parameters the for subject-specific impacts.

Parameter	Unit	H1391	H1406
Gender		Female	Male
Age	[years]	62	94
Height	[m]	1.65	1.73
Body mass	[kg]	59.0	83.9
Measured impact velocity	[m/s]	3.1	3.1
Rotational impact velocity	[1/s]	4.13	4.15
Inertia I_Z_ with respect to foot point*	[kg*m^2^]	12.9	19.6
Specimen mass, pendulum	[kg]	30.56	44.35
Rotational energy, pendulum	[J]	104.1	169.0
Greater trochanter soft tissue thickness	[mm]	32	31
Femoral neck aBMD	[g/cm^2^]	0.694	0.697
T-score	[]	-0.91	-0.88

*based on CAD models with masses and locations measured for each experiments.

### Data collection

Two active infrared-emitting markers were recorded throughout the fall using a motion capture system (Optotrak Certus, Northern Digital Inc., Waterloo, ON, Canada). These markers together with the translation-fixed foot point formed a rigid body that was used to track the motion of the impacted leg over time and estimate the position of the whole specimen during the fall phase.

The impact force was measured using a modified six-axis force plate (FP4000-15, Bertec Corporation, Columbus, OH, USA). The signals were sampled at a frequency of 10 000 Hz using a strain gauge data acquisition system with parallel sampling capability (Chassis: NI SCXI 1000; Amplifier: NI SCXI 1520; terminal box: NI SCXI 1314, National Instruments, Austin, TX, USA). The calibration error for the normal direction was below 1%. Linearity was tested up to 15 kN under quasi-static loading (R = 0.9998). The time to peak force was measured from the first rise in force above 50 N to the time of maximum force.

Two high-speed cameras (Phantom v12, Vision Research, Wayne, NJ, USA) were used to track 8 pelvis markers and 6 soft tissue markers during the impact (Fig 2). To attach markers to the bone all soft tissues were removed from the marker site. The bone surface was subsequently cleaned with ethanol before the markers were glued to the bone surface using a commercial adhesive (Super glue, Gorilla Glue Company, Cincinnati, OH, USA). Markers on the soft tissue surface were glued without cleaning the surface. Both cameras had a resolution of 1200 x 800 pixels (0.3 mm/pixel), a frame rate of 5000 Hz, and an exposure time of 100 μs. Reconstruction of 3D marker positions from camera images was achieved by direct linear transformation [51] using a calibration frame with 11 known marker locations. The frame was oriented in accordance with the orientation of the pelvic inlet during impact. The in plane accuracy of the reconstruction was 0.07 mm in local X and 0.08 mm in local Y. The out of plane reconstruction error was 0.44 mm. Tracking of the distance between two points of a rigid body, that was undergoing a 3D motion, resulted in a standard error of 0.2 mm. A third high-speed camera (Phantom v9, Vision Research) was used to record the overall experiment at a frame rate of 200 Hz; this video was used for qualitative purposes only. Synchronization of the force transducer, high speed cameras, and Optotrak system was achieved using an electrical contact trigger.

### Data analysis

Pelvic deformation was defined from four marker pairs (passive spherical markers, 4 mm diameter; CTMark, Suremark®, Simi Valley, CA, USA) (Fig 2). Two markers were attached close to the pubic symphysis to measure cartilage deformation (C_PS_) across this hip joint. A second marker pair was affixed to the pelvic brim and was used to measure pelvic deformation between the hip joints (C_LR_). Two markers were attached to the iliac wings, close to the sacroiliac joints, and were used to evaluate the compression across these joints and the sacrum (C_SIJ_). Finally, two markers were attached to the anterior superior iliac spines to measure the compression (C_ASIS_) out of the plane of the pelvic inlet. The stiffness of the pelvic region was calculated as the impact load divided by the displacement of the soft tissue marker positioned over the greater trochanter (ST1, Fig 2). A low load range stiffness (k_ST,1_) from 300–1000 N was calculated, to allow for comparison with low drop height fall studies [24, 40]. A high load range stiffness (k_ST,2_) was calculated based on the force-displacement response from 40–90% of peak load.

A planar x-ray system (Mobile 100–15, General Electrics, Boston, MA, USA) was used to take pre- and post-impact x-ray images. The x-ray images were inspected for potential fractures by an orthopaedic surgeon (PG). Furthermore, the pelvis and femurs were visually inspected after the experiment and the pelvis was manually manipulated for its structural integrity by pressing medially on the greater trochanters.

## Results

**Fig 4:**
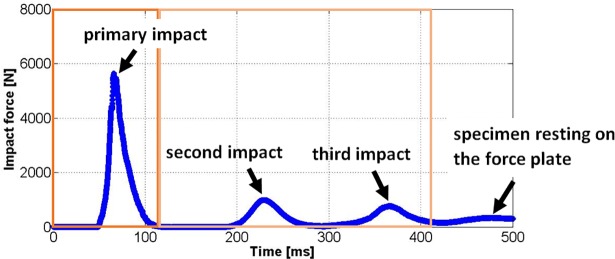
Force-time trace for specimen H1391. Evaluation of the motions, deformations and loads focuses on the primary impact.

**Fig 5:**
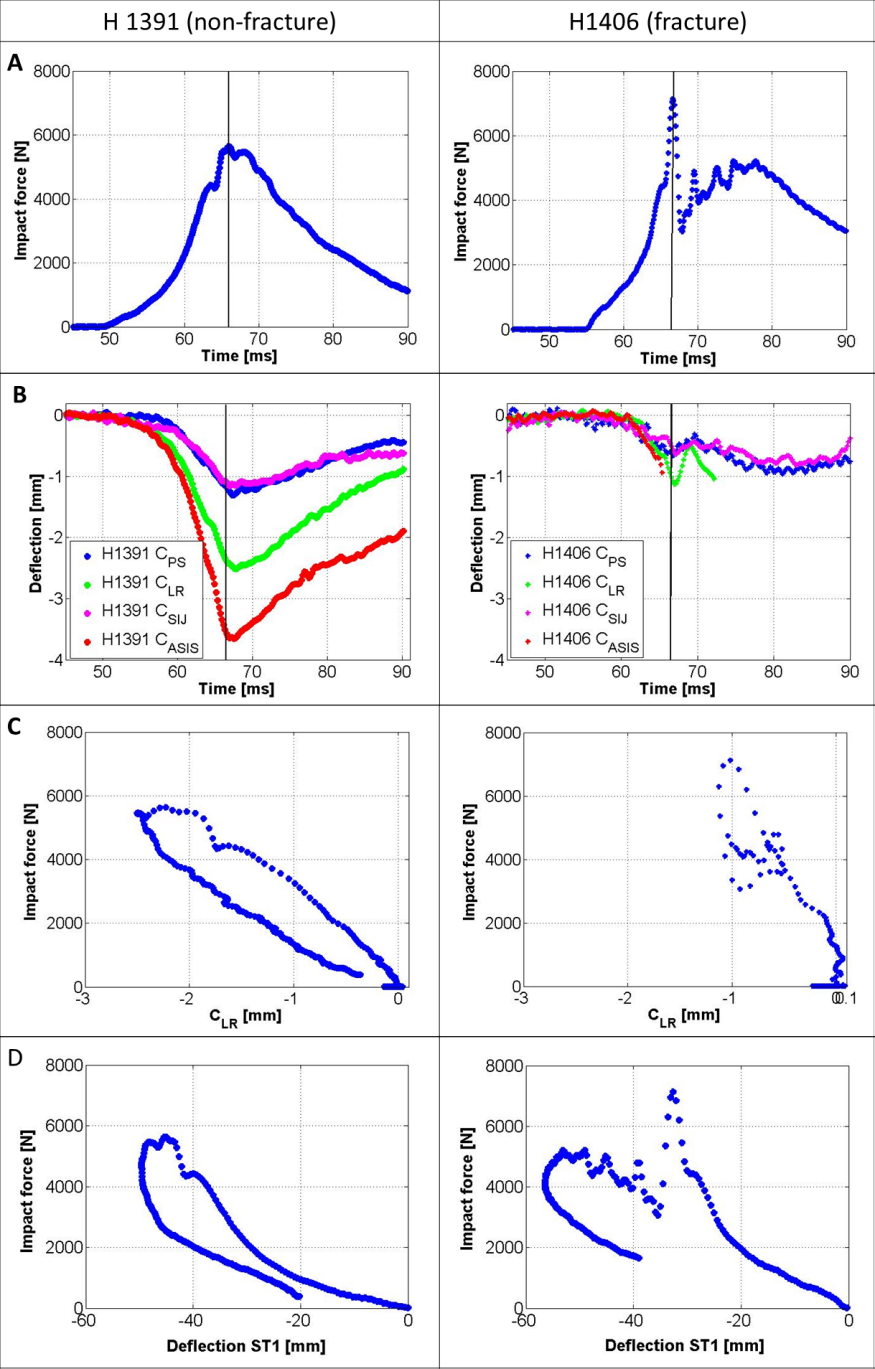
Impact kinetics. (left) Specimen H1391 (female, 60 year, 170 cm, 59 kg, 34 mm soft tissue over the greater trochanter, 107 J impact energy). (right) Specimen H1406 (male, 94 year, 173 cm, 84 kg, 31 mm soft tissue over the greater trochanter, 163 J impact energy). A: primary impact load over time; B: pelvic deflection measured in four locations over time; C: Impact force over pelvic brim deflection, showing the rigidity of the pelvis for both specimens; D: Impact force over the deflection of soft tissue Marker ST1, placed close to the contralateral greater trochanter.

Two specimens, one male and one female, were tested to evaluate the protocol. Prior to testing, specimen H1406 had a broken coccyx and a cut right sacrospinous ligament. Specimen H1391 had a partially fused lumbar disc and a fused left facet joint between vertebra L5 and the sacrum. Three distinct peaks were recorded for specimen H1391 (Fig 4) and two for specimen H1406. The following analysis of the fall simulation focuses on the primary impact, as the event that is most likely to lead to a fracture. A real time video of the impact (H1391) can be found in the supplementary material. The force-time trace of the primary impact (Fig 5) shows a non-linear increase of force, which was recorded for both specimens. Peak impact loads were 5641 N and 7132 N for specimen H1391 and H1406, respectively. The measured time to peak load was 16.3 ms for specimen H1391 and 11.4 ms for specimen H1406. After peak load was reached, specimen H1391 exhibited gradual unloading over 35 ms. In contrast, the load for specimen H1406 dropped sharply within 2 ms from 7132 N to about 3000 N and a second and slower loading phase up to 5211 N was recorded. After reaching this second peak, the specimen was unloaded gradually. The maximum pelvic brim deflection (Fig 5, C_LR_, green curve) was -2.5 mm for specimen H1391. For specimen H1406 a first peak of -1.1 mm was recorded, corresponding to the peak load. A higher deformation was measured during the second loading event. The deflections at the pubic symphysis (C_PS_) and the sacroiliac joints (C_SIJ_) were about half of the deflection at the pelvic brim for both specimens. The deflection of ASIS markers were higher than for the pelvic brim, but could only be fully evaluated for specimen H1391, because for specimen H1406, soft tissue obstructed marker visibility for marker ASIS2 during part of the impact.

Specimen H1391 showed higher pelvic deflections at equivalent loads compared to specimen H1406 (Fig 5). High speed videos are provided in the supplementary material. For the load over deflection of the contralateral soft tissue marker (ST1), specimen H1391 had a higher deflection up to peak load compared to specimen H1406, but for specimen H1406 a higher total marker deflection was recorded.

Measured high load range stiffness values (k_ST,2_), for the impact load over the deflection of marker ST1, were 5–6 times higher than low load range stiffness values (k_ST,1_) (Table 2).

**Table 2:** Effective pelvic stiffness.

	Force range	H1391	H1406
k_ST,1_ [N/mm]	300–1000 N	50.7	79.5
k_ST,2_ [N/mm]	40–90% of peak load	245.6	473.3

**Fig 6:**
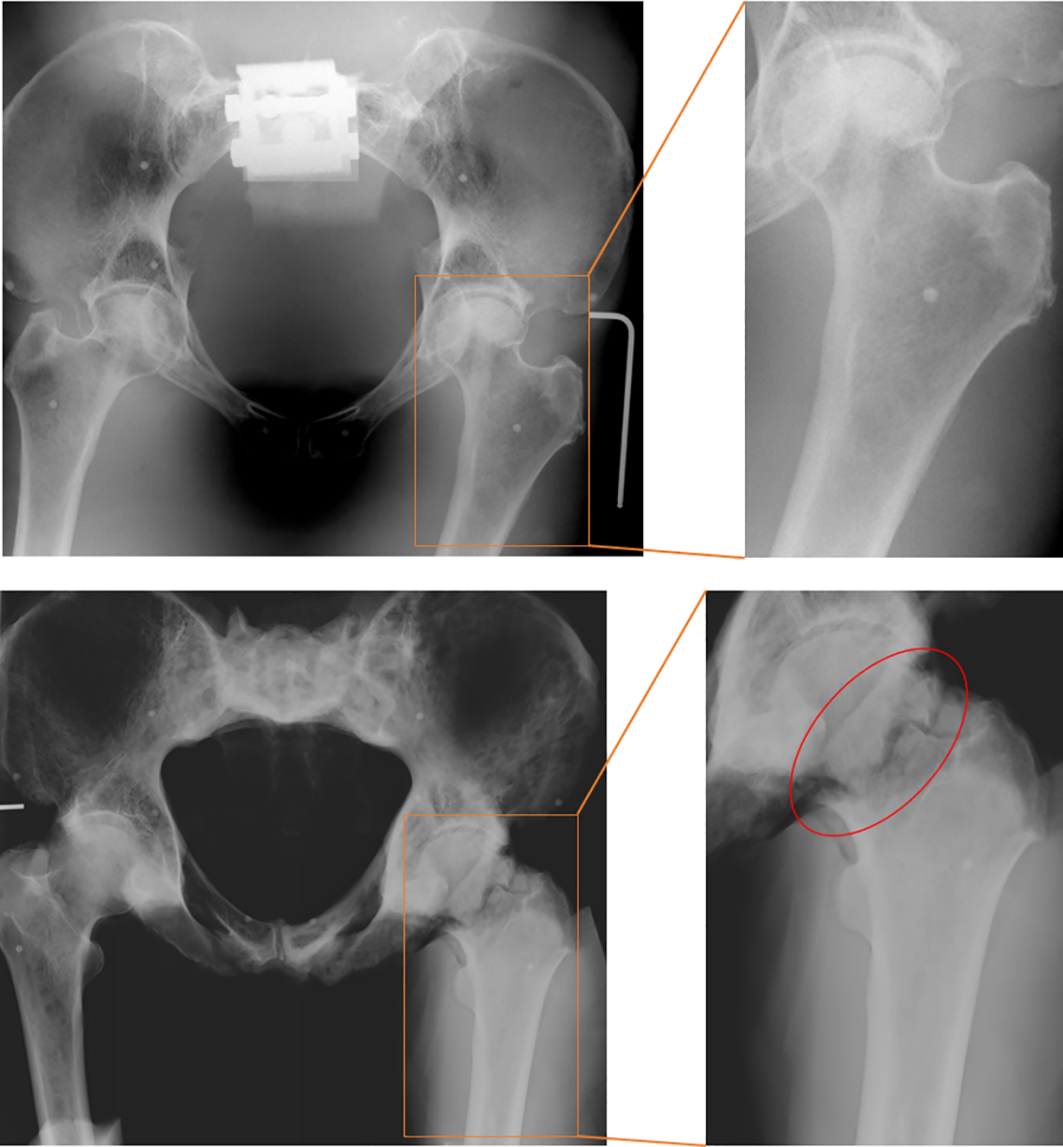
Fracture evaluation. Post-test X-ray of specimen H1391 (top) and H1406 (bottom). (left) pelvic anterior-posterior view showing the pelvis and both femurs. (right) Zoomed view of impacted femur (left femur). The Circled area indicates the fracture site for specimen H1406. No fracture was visible for specimen H1391.

Post-test x-rays showed a fracture of the impacted femur for specimen H1406 (Fig 6). The observed fracture was an intracapsular femoral fracture, comparable to clinically observed fractures. [52–54] The soft tissue over the greater trochanter had a small tear on both specimens. No further damage to bone, cartilage, ligaments, or soft tissues due to the impact was detected for either specimen.

## Discussion

The goal of our study was to create a biofidelic sideways fall simulator that represents a fall from standing with an impact to the greater trochanter. We suspected that a biofidelic simulation, incorporating a full pelvis and both femurs, with inertia-driven boundary conditions and realistic body posture could result in femoral fractures as opposed to pelvic fractures reported in cadaveric full pelvic impact studies. [33–35, 55] Moreover, we suspected that testing with such a protocol would result in fracture and non-fracture outcomes as only a minority of real-world falls result in hip fractures. [52, 56] Of the two specimens tested, one sustained a femoral fracture, suggesting that the method has the potential to allow for discrimination between subjects at high and low risk of sustaining a hip fracture. The fracture is comparable to clinically observed fractures, [53] and therefore meets our goal of producing clinically relevant hip fractures in a sideways fall simulation. In contrast to other cadaveric studies, that resulted purely in pelvic fractures, [33, 35, 55] the specimens in the current study were loaded solely based on their mass moment of inertia and velocity without further constraining the pelvis. The load acting in such a moving impacting massive spring decreases with the distance from the impact surface, therefore reducing the risk of obtaining pelvic fractures. Furthermore, the alignment of the pelvis in this study was different compare to previous studies. [33, 35, 55] These studies simulated lateral vehicle impacts. Therefore, the pelvis was aligned with both hip centres in line with the loading direction. The present protocol rotated the pelvis 15° in the coronal plane to simulate a fall from standing with an upper body flexion away from the ground, as can be observed in volunteer falls [38, 43] and video data of real-world falls. [57, 58] This rotation resulted in a pelvic alignment that favours the load transfer through the sacrum instead of the pubic symphysis, thus decreasing the likelihood of rami fractures which are the most common type of pelvic fractures in cadaveric studies testing full pelvises under lateral impact. [33–35, 55] Ground contact of the knee was observed at about 100 ms past trigger, which was approximately 30 ms later than peak force during primary impact. As a result, the mass moment of inertia of the lower limb additionally contributed to loading the femur.

Another expectation was that specimen loading would be non-linear and that the protocol would allow us to evaluate differences in pelvic compliance and their effect on femur loading. Specimen H1391, which did not fracture, allowed us to observe the complete force impulse that this specimen had to withstand due to the simulated fall. Specimen H1406 may have experienced a higher peak impact load, had it not fractured during the impact. The measured stiffness in the low load range was comparable to previously published low drop height stiffness values. [24] However, the high load range stiffness was 5 to 6 times higher than low force range stiffness. In this load range, the soft tissue over the greater trochanter was likely almost completely compressed. As a result, the high force range stiffness is close to pelvis stiffness values that have been reported to be in the range of 201–423 N/mm. [55] Consequently, extrapolation of the load experience during as fall to the side based on experiments with lower peak loads could lead to inaccuracies. At equal load levels, specimen H1391 deformed more compared to specimen H1406, indicating that a larger amount of energy was stored in the pelvis. Interestingly, specimen H1391, which experienced a larger pelvic deformation, did not fracture, while specimen H1406 did.

Although our system represents more biofidelic loading conditions than previously reported cadaveric sideways fall studies, this study has several limitations. Firstly, soft tissues were represented by one homogeneous surrogate material instead of heterogeneous human tissues. Native tissue would not have maintained its original properties [59, 60] and its structural integrity would have been compromised when affixing markers. Further, the input energy would have been less comparable between specimens and the variance in composition and mechanical properties may have prevented assessment of the effect of the bone structures and soft tissue geometry on the impact force and injury outcome. We aimed to balance the inclusion of some variability (such as that in bone and soft tissue geometry) and exclusion of other sources of variability (such as native post-mortem soft tissue). A second limitation was the assumption of total muscle passivity, which only represents one extreme of the spectrum of possible muscle activation patterns. Little information is available with respect to muscle activation during falls. In general, elderly people have longer reaction time and weaker muscles. Therefore, neglecting muscles activation was chosen over assuming a particular muscle activation without experimental evidence. A third limitation is that only the lower extremities and pelvic region up to approximately the navel were represented in the sideways fall simulation. This assumption was based on the posture observed in published images and videos of falls. [38, 58] The observed protective motion to avoid head impact was to bend the torso and with it the spine laterally to increase the distance between the head and the ground. This posture together with muscle passive assumptions requires high deflection and deformation of the upper extremities and the spine in order to recruit their masses for load introduction to the hip. Other limitations include the artificial increase in leg length, which resulted in an error in the inertial representation at the lower limbs. This leg length increase was necessary to reach velocities higher than 3.0 m/s, with our pendulum that was released with flexed legs. Further, the load at the hip joint could not be measured without damaging the specimens. Therefore, only the impact load at the ground was measured. Compared to reported femoral strength values our peak impact loads were relatively high. [12, 30, 31, 61] However, the measured load is the load acting between the impact surface and the soft tissue, which is not equal to the load transferred through the femur. A study with a surrogate system reported the load at the femoral neck to be 20% lower compared to the load between the soft tissue and the impact surface. [29] With the specimen in motion in our study, the difference between the force acting on the ground and the force acting on the femur might even be larger due to inertial effects. Taking this into account, the femoral loads were likely within the range of typical femoral strength values. [12, 30, 31, 61] In summary, the presented protocol provides an ex vivo method to test cadaveric pelvis-femur specimens with different geometries under controlled impact configurations. This allows for inter-specimen comparison to identify biomechanical factors influencing impact loads and injury outcomes in a fall from standing, which cannot be tested with volunteers. Further, the protocol provides a biofidelic method that could be used to test different impact configurations, preventive measures, and to validate computational models.

## Conclusion

To our knowledge, this is the first experiment to create a clinically relevant femoral fracture and a non-fracture outcome with the same setup using impact energies and velocities representative of a fall to the side from standing height. We tested cadaveric pelvis-femur specimens embedded in a soft tissue surrogate material with mechanical properties representative of a mixture of human fat and muscle tissue. The results showed a non-linear force-time profile, which implies that extrapolation based on less severe loading conditions may not be accurate. The fall phase of our protocol was controlled with respect to specimen alignment and motion, while the specimens were allowed to freely move at the time of impact. This method could be used to discriminate between high and low risk individuals and to investigate the role of different pelvic geometries and body shapes, as well as body postures and impact velocities.

## Supporting information

S1 Methods and Alignment Additional information on energy calculation, lower limbs design, alignment, and soft tissue moulding. (PDF)
