# Supplementary material for: A novel sideways fall simulator to study hip fractures ex vivo
Source: PLoS One. 2018 Jul 24;13(7):e0201096. doi: 10.1371/journal.pone.0201096 (PMC6057661; doi:10.1371/journal.pone.0201096)
Supplement: S1 Methods and Alignment — (PDF) [file pone.0201096.s001.pdf]

Energy calculation

We converted translational impact energy to rotational energy for the inverted pendulum (S1 Fig). The energy input and a greater trochanter impact velocity of 3.0 m/s were used as target to adjust the pendulum's inertia. The pendulum's mass (52 % body mass), including lower limb constructs, soft tissue, cadaveric parts and rollers, that was necessary to achieve the desired inertia was much higher than the effective mass (38 % body mass) of a translational sDOF model. The required mass corresponded to the mass of lower limbs and abdominal region based on literature. (1)

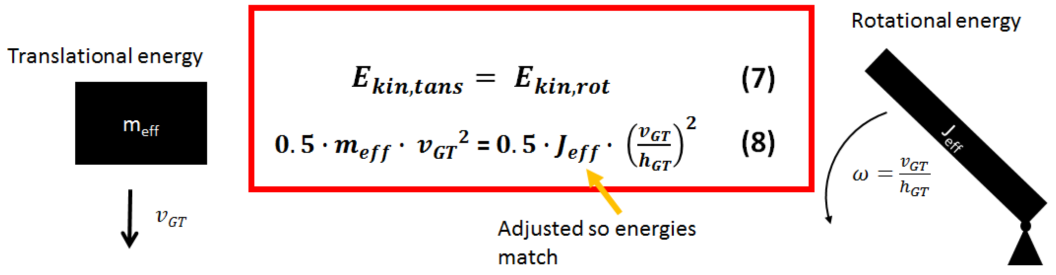

S1 Fig translation from sDOF energy input (translational energy) to pendulum energy input (rotational energy). The pendulum height was fixed for the experiment and the greater trochanter velocity was kept constant. As a result, the pendulum inertia was the parameter that was adjusted depending on the theoretical effective mass of the specimen.

Lower limb construction

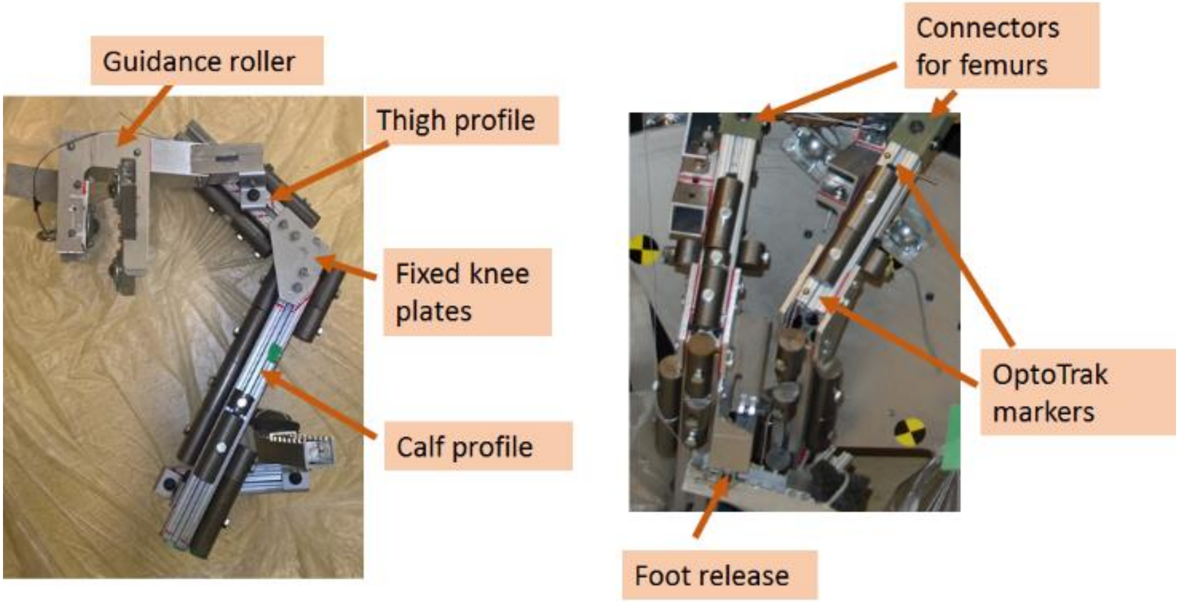

S2 Fig lower limb design. (left) left lower limb construction with masses for a heavy specimen. (right) lower limb constructions in the setup with a specimen connected at the top end and OptoTrak markers fixed to the left leg.

Aluminium profiles for thigh and calf with stiff plates at the knee that fixed the angle. Cylindrical masses to adjust the weight according to subject mass

Mass adjustment

S1 Table target masses for body segments.

Body segment	Adjusted mass	Literature (1)
Foot and Calf	6 % body mass	6.4 % body mass
Lower thigh (leg construction)	4% body mass	10 % body mass
Upper thigh (bone and gel)	6% body mass	
Abdomen (up to naval)	20 % body mass	Based on target impact energy

Grey shaded rows highlight the leg construction. Orange shaded rows highlight the cadaveric specimen and soft tissue surrogate.

Target alignment

A triangle between the foot point (F), knee point (K) and a point created by projecting the estimated impact point onto the femoral shaft axis (H), was used to define a lower limb triangle for both legs (S3 Fig, A). Segment length ratios (1) and the calf angle (2) were taken from literature and used to calculate hip flexion and knee flextion (Figure 3 B).

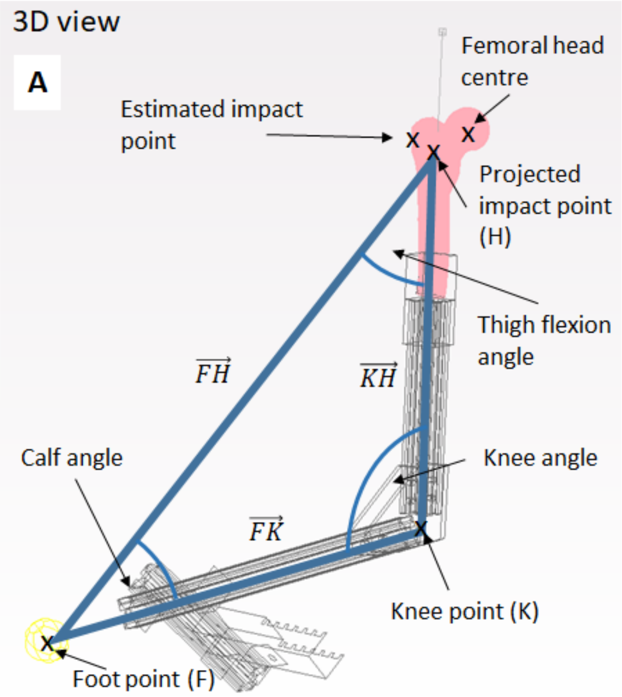

B Impacted femur	[°]	Ref
**Hip flexion angle ***	37.6	
**Calf angle**	33.4	[2]
**Knee angle***	109	
**Internal rotation****	15	
**Femur anteversion angle**	-13	[3]
**Contralateral femur**	[°]	Ref
**Hip flexion angle ***	37.6	
**Calf angle**	33.4	[2]
**Knee angle***	109	
**Internal rotation****	0	
**Femur anteversion angle**	-13	[3]
**Pelvis**	[°]	Ref
**Pelvis rotation about Z****	15	
**Pelvic tilt**	12	[4]
* Calculated value, ** chosen value		

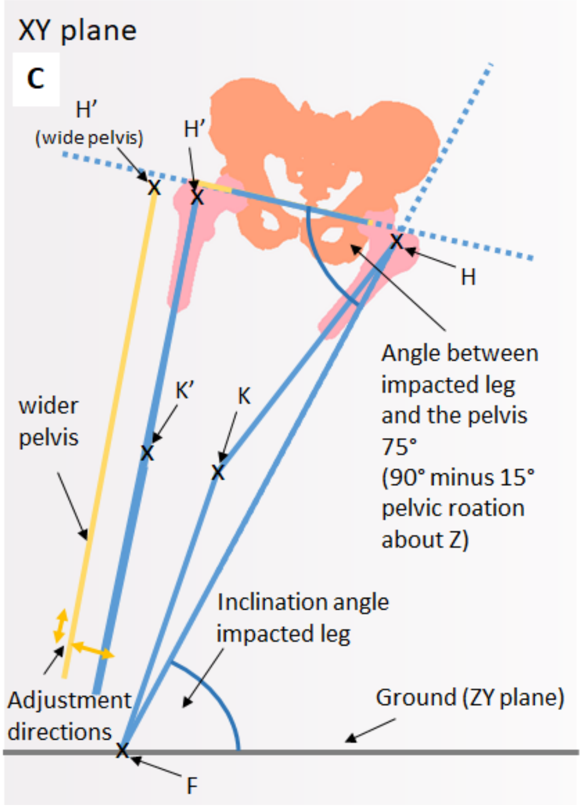

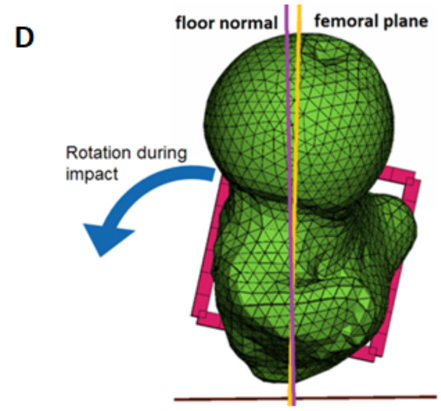

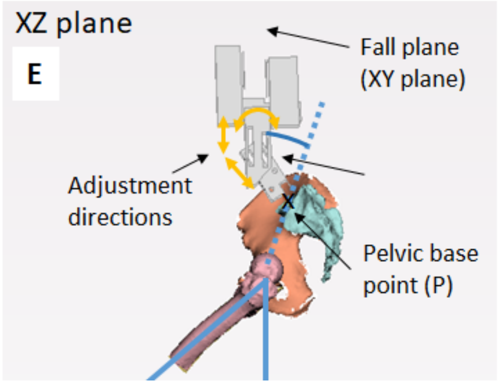

S3 Fig Body posture and detailed alignment. (A) Schematic triangle for lower limb angle calculation. (B) Target angles for both limbs and the pelvis. (C) schematic of angles and adjustment options of the specimen in the setup (D) pelvic tilt and pelvic roller adjustment options (E) femoral neck alignment right before impact

A femoral anteversion angle of 13 ° was chosen. (3) To lift the knee off the ground a combination of internal rotation and adduction was applied. A 2 ° bigger internal rotation than femoral anteversion

angle was chosen to ensure that the femoral neck passed through a vertical alignment with the ground during the impact (S3 Fig, D). The contralateral limb was assumed to mirror the impacted limb’s thigh flexion and knee angle, but not the internal rotation and adduction.

Both femoral head centres were aligned to be in the same XY plane (S3 Fig, E). The pelvic tilt was set to 12 °. (4) A fall alignment with the upper body flexed only in the same fall plane was assumed. Therefore, a pelvic rotation in the coronal plane of 15 ° was selected for this study. This angle was assumed to be one part of the upper body to ground angle (2) which is a combination of pelvic rotation with respect to the ground and lateral spine bending. The upper body superior to the base of sacrum was not modelled.

To confirm the experimental alignment and position of the specimen in the rig, a virtual experimental rig was created in MATLAB (Mathworks, Natick, MA, USA). Digitized points and markers in combination with CT based marker locations and bony landmarks were used to measure the position of the femurs and pelvis with respect to the setup and with respect to each other (S4 Fig).

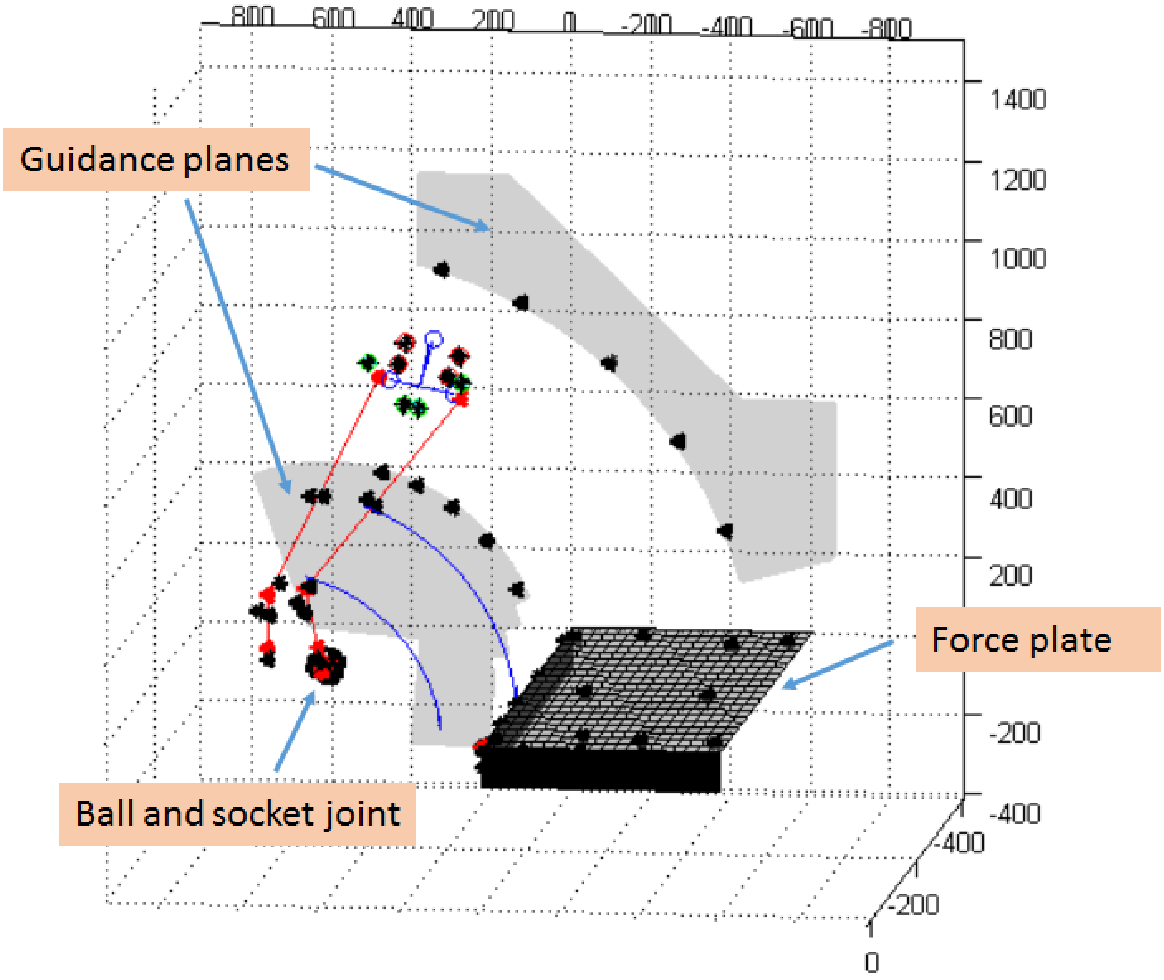

S4 Fig Virtual experimental setup. Virtual reconstruction, used to calculate specimen alignment prior to releasing the pendulum. *Black points*: digitized points; *red elements*: lower limb reconstruction; *blue lines above the legs*: pelvic alignment reconstruction based on digitized markers; *blue circle segments*: tracked active markers over the fall phase.

Adjustable angles were iteratively changed until they were within 5 ° of the target value prior to releasing the specimen (S2 Table). Subject specific differences in the distance between the femoral heads and the height of the pelvis required some adjustment options to position specimens into similar alignments. Angles that had not to be adjusted between specimens were thigh flexion, knee and calf angle of each lower limb, thigh internal rotation angle for both lower limbs, and thigh adduction of the impacted limb. Thigh adduction of the contralateral leg was adjusted based on the distance between the femoral head centres of the specimen. The pelvic rotation about the Z-axis was also adjusted with a slider at the pin release between impacted and contralateral leg. The pelvic roller was adjusted

depending on pelvic height and inclination angle of the base of the pelvis. Therefore, two translational and one rotational degrees of freedom in the sagittal plane of the pelvis were adjustable. Degrees of freedom for adjustment are shown in yellow in Figure 3 (C and E).

S2 Table Measured body posture and alignment.

	Target angles	H1391	H1406
**Impacted femur**			
Flexion angle [°]	37.6	37.3	37.1
Calf angle [°]	33.4	33.8	33.8
Knee angle [°]	109	108.9	109.1
Internal rotation [°]	15	14.8	14.65
Femur Anteversion angle [°]	13	10.8	12.3
**Contra femur**			
Flexion angle [°]	37.6	37.2	37.4
Calf angle [°]	33.4	32.5	32.2
Knee angle [°]	109	111.3	111.4
Internal rotation [°]	0	-1.1°	-1.2
Femur Anteversion angle [°]	13	7.0	7.9
**pelvis**			
Pelvic rotation about Z [°]	15	13.7	15.2
Pelvic tilt [°]	12	12.4	7.1

Soft tissue surrogate

During dissections the material over the greater trochanter was observed to be a combination of skin, adipose tissue, fascia, and tendon attachments, which supported the choice of a surrogate material with properties in between muscle and adipose tissue.

Custom mould shapes, based on a shapes database (SizeUSA, [TC]^2 Labs, Apex, NY, USA) were created for each specimen to represent the desired soft tissue geometry, mass and inertia. The following steps were performed to mould the soft tissue surrogate around the specimen:

1. CT segmentation of femur and pelvis geometries
2. Selection of a database shape based on the bone geometry, BMI, mass, height, and desired soft tissue thickness for each subject.
3. Positioning of the segmented bones into fall alignment
4. Positioning the soft tissue shape around the specimen and morphing it into fall alignment (S5 Fig, image 1)
5. Fine tuning of the shape for target inertia and mouldability. E.g. The upper thighs were connected.
6. Virtual mould shape creation (S5 Fig, image 2)
7. Physical mould shape creation in polystyrene foam (S5 Fig, image 3)
8. Ballistic gel mixing
9. Wrapping of ballistic gel soaked fabric patches around the distal femurs and the square tubes
10. Application of a ballistic gel soaked fabric patch on the right posterior side of the mould shape
11. Positioning of the specimen in the mould shape in fall alignment
12. Casting the gel around the specimen. The gel was at a temperature of 30 degrees Celsius when cast around the specimen.
13. Storage of the mould and specimen in at 4 degrees for 38 hours
14. Demoulding and storage at room temperature for 30 hours (S5 Fig, image 4)
15. Preparation of fiducial markers on bone and soft tissue surrogate with dark backgrounds (S5 Fig, image 4 and 5)
16. Dynamic testing 68 hours after moulding the gel around the specimen

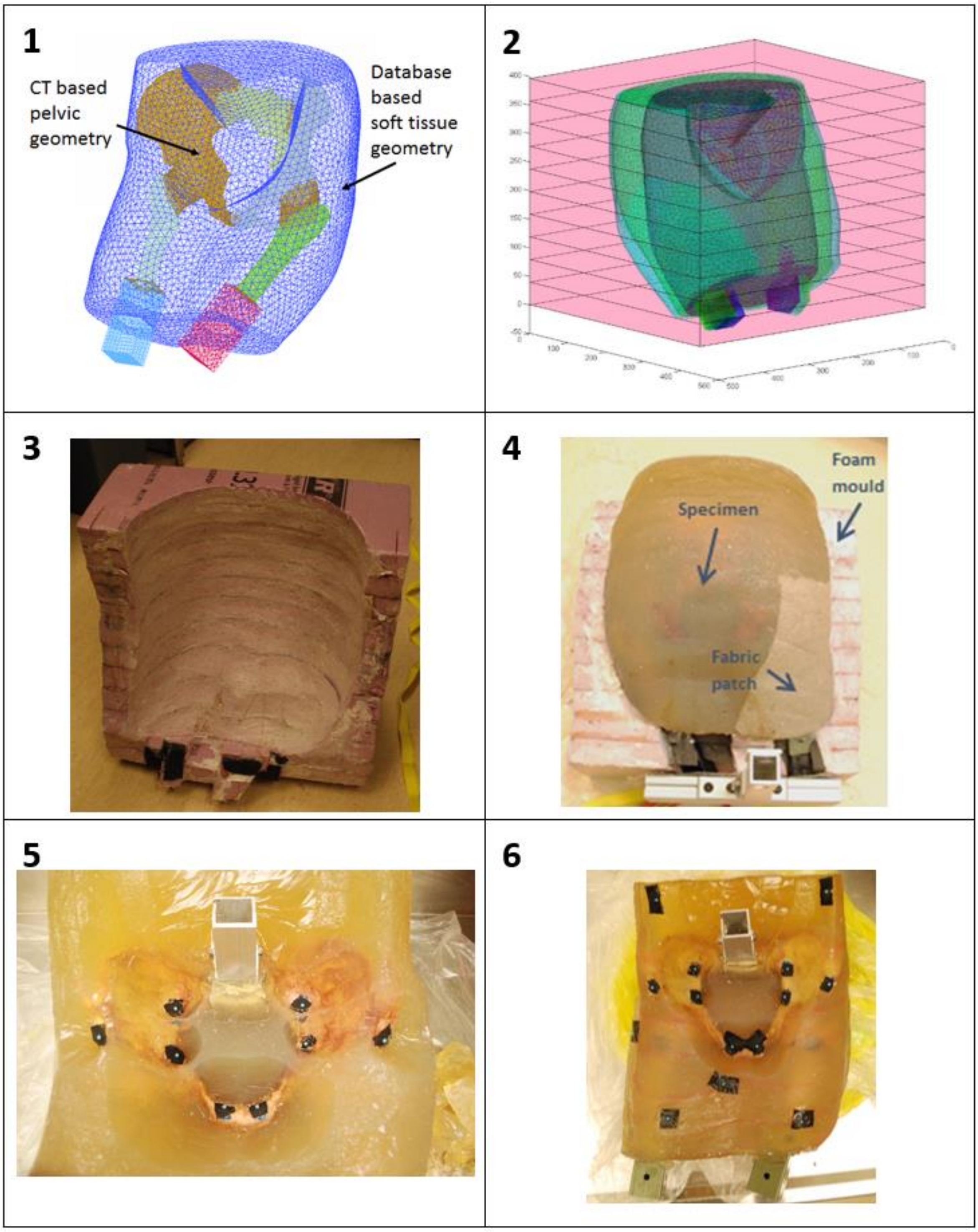

S5 Fig Soft tissue surrogate moulding steps: 1) database shape with CT-based bone geometry; 2) virtual model for mould creation; 3) physical foam mould, posterior part; 4) specimen while demoulding, still in the anterior part of the mould. 5) demoulded soft tissue with ballistic gel removed from marker sites for marker visibility; 6) demoulded specimen with spherical markers attached. Black tape was added to improve contrast for fiducial markers.

References

1. Dempster WT, Gaughran GRL. Properties of body segments based on size and weight. American Journal of Anatomy. 1967;120:33-54.

2. van den Kroonenberg AJ, Hayes WC, McMahon TA. Hip impact velocities and body configurations for voluntary falls from standing height. Journal of biomechanics. 1996;29:807-11.

3. Reikerås O, Bjerkreim I, Kolbenstvedt A. Anteversion of the acetabulum and femoral neck in normals and in patients with osteoarthritis of the hip. Acta orthopaedica Scandinavica. 1983;54:18-23.

4. Boulay C, Tardieu C, Hecquet J, Benaim C, Mouilleseaux B, Marty C, et al. Sagittal alignment of spine and pelvis regulated by pelvic incidence: standard values and prediction of lordosis. European Spine Journal. 2006;15:415-22.
